# Suppression of Diet-Induced Hypercholesterolemia by Turtle Jelly, A Traditional Chinese Functional Food, in Rats

**DOI:** 10.1155/2012/320304

**Published:** 2012-10-31

**Authors:** Jian-Hong Wu, Qing-Hua Wang, Fan Li, Yuan-Lan Shu, Chi-On Chan, Daniel Kam-Wah Mok, Shun-Wan Chan

**Affiliations:** ^1^State Key Laboratory of Chinese Medicine and Molecular Pharmacology, 518057 Shenzhen, China; ^2^Department of Applied Biology and Chemical Technology, The Hong Kong Polytechnic University, Hong Kong; ^3^Food Safety and Technology Research Centre, Department of Applied Biology and Chemical Technology, The Hong Kong Polytechnic University, Hong Kong

## Abstract

Consumption of functional foods for lowering serum cholesterol has globally gained acceptance by the general public. Turtle jelly (TJ), also called gui-ling-gao, is a popular traditional functional food in southern China. The hypocholesterolemic effect of consuming TJ was investigated in rats fed with normal diet, high-cholesterol diet or high-cholesterol diet supplemented with simvastatin (3 mg/kg bw per day, p.o.) or TJ (3.3 or 10 mL/kg bw per day, p.o.) for 30 days. TJ markedly reversed the increased serum total cholesterol, increased high-density lipoprotein, and decreased high-density lipoprotein induced by hypercholesterolemic diet with a dose-dependent improvement on the atherogenic index. It also demonstrated good hepatoprotective function by reducing fat depositions and overall lipid contents in the liver and increasing the activities of hepatic antioxidative enzymes. The blunted nitric oxide/endothelium-mediated aortic relaxation in rats fed with hypercholesterolemic diet was partially restored after TJ consumption. It is postulated that the hypocholesterolemic effect is the primary beneficial effect given by TJ; it then leads to secondary beneficial effects such as vasoprotective and hepatoprotective functions. The results revealed that TJ could block the downregulation of LDLR and PEPCK and upregulation of PPAR**α** mRNA and protein expressions in the livers of rats fed with hypercholesterolemic diet.

## 1. Introduction

Hypercholesterolemia is a dominant risk factor for the development and progression of atherosclerosis and is closely related to cardiovascular diseases (CVD) [1]. The modern lifestyle of high-cholesterol diet and inadequate physical activity contributes to hypercholesterolemia which increases the prevalence of CVD. Diet high in cholesterol is a major environmental contributor to the metabolic disturbance of lipoprotein which is closely tied to atherosclerosis, a leading cause of death in the developed countries. It is generally believed that reduction of total cholesterol and low-density lipoprotein cholesterol (LDL) reduces atherosclerosis in animals and clinical cardiovascular events in humans [2, 3]. Although drugs are available clinically for handling hypercholesterolemia, the consumption of functional foods or dietary supplements for lowering serum cholesterol and risk of CVD has gained enormous global acceptance over the years by the general public [4, 5].

Turtle (Tortoise) jelly, also called gui-ling-gao in Chinese, is a popular traditional functional food in southern China and Hong Kong. Traditionally, it is prepared from tortoise (*Chinemys reevesii* Gray) shell and various Chinese medicinal herbs such as *Smilacis Glabrae Rhizoma*, *Millettiae speciosae Radix*, *Mesona chinensis *Benth, and *Lonicerae Japonicae Flos*. Currently, turtle jelly is prepared by more or less the same herbal components. Since the traditionally use tortoise species, *Chinemys reevesii* Gray, is an endangered species, people use other nonendangered farm grown tortoise species, such as *Ocadia sinensis*, to obtain the tortoise shell. Turtle jelly is said to possess multiple health-related beneficial effects such as clearing toxicants from the blood, protecting the liver, minimizing the effects of damp-heat, improving skin disorders and nourishing “yin” [6]. However, there is no solid evidence for these anecdotal health-benefit claims. 

In our previous study, turtle jelly was demonstrated to have antioxidant capacity and ability to remove endogenous free radicals *in vitro*. In order to further verify the anecdotal health-benefit claims of turtle jelly *in vivo*, a study was launched to investigate the effects of high-cholesterol diet in rats with or without supplementation of turtle jelly on the serum lipid profiles [triglycerides, total cholesterol, high-density lipoprotein (HDL), low-density lipoprotein (LDL)], levels of fat depositions in liver tissues, and changes in the activities of key hepatic antioxidative enzymes. Further investigations on the effects of turtle jelly on endothelium-dependent vasorelaxation in rat-isolated aortic rings and the gene and protein expressions of low-density lipoprotein receptor (LDLR), phosphoenol pyruvate carboxykinase (PEPCK), peroxisome proliferation-activated receptors *α* (PPAR-*α*), and 3-hydroxy-3-methyl-glutaryl-CoA reductase (HMGR) in the livers were carried out so as to understand the molecular mechanism of turtle jelly in the hepatic system.

## 2. Materials and Methods

### 2.1. Preparation of Turtle Jelly Sample Solution and Chemicals

Turtle jelly was obtained from Hoi Tin Tong Fresh Gui Ling Gao Company Limited (Hong Kong SAR, China) and it was stored in dark at 4°C. The turtle jelly was concentrated by heating to 100°C until the volume was reduced to one-eighth of the original volume. The resultant solution of turtle jelly was centrifuged (at 625 ×g, 10 min) and filtered. The final filtrate was used as the testing stock solution. The concentration of the concentrated turtle jelly solution (stock solution) was determined by lyophilizing 1 mL of the solution in a freeze drier (Labconco, FreeZone 6). The dry weight of 1 mL concentrated turtle jelly solution was 0.0686 g. Therefore, the concentration of the concentrated turtle jelly sample solution was 68.6 mg/mL. The tested dosages of turtle jelly were 3.3 and 10 mL/kg bw per day, which were equal to 0.23 and 0.69 g/kg bw per day, respectively. The selected dosages of turtle jelly (3.3 and 10 mL/kg bw per day) were equivalent to one and three times of the recommended human daily consumption, respectively. Simvastatin 20 mg tablets (10% of tablet weight, content confirmed by HPLC), a positive control, were purchased from Hangzhou MSD Pharmaceutical Co., Ltd. Phenylephrine, acetylcholine, indomethacin and neostigmine were purchased from Sigma Chemicals Co. (St. Louis, MO, USA). All other chemicals used were of analytical grade.

### 2.2. High-Performance Liquid Chromatography (HPLC) Analysis on Turtle Jelly Sample

Freeze-dried turtle jelly sample (0.1 g) was accurately weighed and extracted twice with 80% methanol (10 mL) for 30 min. The extracted solutions were combined and evaporated to dryness with a rotary evaporator. Before HPLC-DAD analysis, the residue was redissolved in 80% methanol (5 mL) and the sample solution was filtrated through a syringe filter (0.45 *μ*m). Chromatographic analysis was carried out on a C_18_ column (250 mm × 4.6 mm, 5 *μ*m) (Alltima, Grace) at 25°C using an Agilent 1100 liquid chromatography system equipped with a quaternary solvent deliver system, an autosampler and a DAD system. The detection wavelength was 254 nm. The gradient elution of the mobile phase consisting of (A) 1.0% (v/v) formic acid and (B) methanol is as follows: 90% (A) at 0 min; from 90% to 50% (A) at 0–50 min and from 50% to 10% (A) at 50–60 min. 10 min reequilibrium was allowed between injections. The flow rate was 1.0 mL/min and aliquots of 10 *μ*L were injected into the HPLC. To identify components in the turtle sample, an Agilent MSD Trap VL module mass spectrometer was connected to the Agilent 1100 HPLC instrument via an electrospray ionization ESI interface. The LC effluent was introduced into the ESI source in a postcolumn splitting 5 : 1. Ultra high-purity helium was used as the collision gas, while high-purity nitrogen was used as the nebulizing gas. The parameters in the negative/positive ion mode were as follows: the heated capillary temperature, 350°C; nebulizer gas, 35 psi, dry gas, 10 L/min. Data acquisition was performed in the full-scan mode form m/z 100 to 1500 for mass spectrometer and with an accumulation time of 200 ms and 7 microscans were averaged per recorded scan.

### 2.3. Animals and Experimental Treatment

Male Sprague-Dawley rats (170 ± 10 g) were supplied by Guangdong Provincial Medical Laboratory Animal Center (Guangzhou, China). Rats were housed under standard conditions (temperature 24 ± 2°C, humidity 60 ± 10%, light from 6 AM to 6 PM) with free access to water and rat chow. After acclimation for a week in this laboratory environment, the rats were randomly assigned to one of the five different experimental groups (each with *n* = 8). These groups were (1) Control: a control group fed with normal rat chow obtained from Guangdong Provincial Medical Laboratory Animal Center (Guangzhou, China) [composition: protein (~14%), fat (~10%), and carbohydrate (~76%)]; (2) HCD: a high-cholesterol diet group fed with high-cholesterol diet, which is a standard rat chow supplemented with 1% cholic acid, 2% pure cholesterol and 5.5% oil, as described elsewhere [4, 7]; (3) SIM: a simvastatin treatment group that received high-cholesterol diet plus simvastatin (3 mg/kg bw per day, p.o.); (4) GL: a low-dose turtle jelly treatment group that received high-cholesterol diet plus turtle jelly (3.3 mL/kg bw per day, p.o.); (5) GH: a high-dose turtle jelly treatment group that received high-cholesterol diet plus turtle jelly (10 mL/kg bw per day, p.o.). The rats were administered with distilled water (vehicle) or their corresponding treatments by oral gavage (20 mL/kg) once every morning for 30 days. At the end of the experimental period, the rats were fasted overnight and killed by cervical dislocation. Blood, livers, and aortas were then collected for further analysis. The experimental protocol was conducted under the animal license issued by the Health Department of the Hong Kong SAR Government and the Animal Subjects Ethics Sub-committee (ASESC no. 05/21) of The Hong Kong Polytechnic University. All procedures were consistent with the *Guide for the Care and Use of Laboratory Animals* published by the US National Institutes of Health and the principles outlined in the Declaration of Helsinki. Every effort was made to limit animal suffering and the number of animals used in this study. 

### 2.4. Analysis of Lipid Levels in Blood Samples

Immediately after cervical dislocation, blood was collected in chilled centrifuge tubes by cardiac puncture and allowed to clot for 2 h at 4°C. The clotted blood was centrifuged (2000 ×g) at 10°C for 10 min to get the serum. Serum was stored at −80°C until the measuring of lipid profiles. Total serum cholesterol, triglycerides, LDL, and HDL were measured by the ALYCON systems using Roche Reagents.

### 2.5. Liver Histopathological Examination and Lipid Content Evaluation

Livers obtained from all experimental groups were first perfused with saline before isolating from the animals. They were washed immediately with saline, blotted dry, and weighed. One lobe of each liver sample was then fixed with 4% paraformaldehyde in 0.1 M sodium phosphate buffer (pH 7.4) overnight at 4°C. After rinsing with water, the samples were dehydrated and embedded in paraffin wax. Then, the tissue was sectioned to about 5 *μ*m in thickness, stained with haematoxylin and eosin and examined under light microscope (400×) and photomicrographs were taken. 

Liver samples (each ~2 g) were dissected and homogenized with 2 : 1 chloroform-methanol mixture (v/v) to a final dilution of 1 : 20 w/v using Ultra-Turrax T-25 homogenizer. After filtration, 10 mL of individual filtrate was added to 2 mL water and the mixture was centrifuged at 900 g for 20 min. The lower phase was dried and its weight was measured [8]. Liver lipid content was expressed as weight of lipid per g of liver. 

### 2.6. Determination of the Antioxidative Enzyme Activities and MDA Content in Livers

To monitor the activities of antioxidative enzymes in the livers from various experimental groups, isolated liver (~1 g) of each rat was weighed and kept at −80°C immediately until homogenization procedures. Each frozen sample was homogenized in ice-cold saline (1/9, w/v) using an ultra-Turax T-25 homogenizer for 10 bursts of 10 s each, separated by a pause of 15 s. Then, the homogenates were centrifuged at 10000 ×g for 5 min (4°C), and the supernatants were collected and stored at −20°C until enzyme activities analysis. Superoxide dismutase (SOD), glutathione peroxidase (GSH-Px), catalase (CAT), and maleic dialdehyde (MDA) were measured using commercial kits (Nanjing KeyGen Biotech. Co., Ltd., China). SOD, GSH-Px, and CAT activities were expressed as U/mg protein. MDA content was expressed as mmol/mg protein. The protein content was measured by bicinchoninic acid (BCA) assay kit (Shenergy Biocolor, Shanghai, China).

### 2.7. Isolation of Thoracic Aortas

At the end of the treatment period, the rats were sacrificed and their thoracic aortas were immediately placed in 4°C Tyrode's solution of the following composition: NaCl 118 mM, KCl 4.7 mM, KH_2_PO_4_ 1.2 mM, NaHCO_3_ 25 mM, glucose 11 mM, CaCl_2_ 2.5 mM, and MgSO_4_ 1.2 mM. The isolated aorta from each animal was cut into three ring segments with all fat and connective tissue removed. One ring from each aortic preparation was used for endothelium-dependent vasorelaxation (aortic ring, ~3 mm), one for *in vitro* nitrite production (aortic ring, ~15 mm) and one for real-time PCR analysis of eNOS (aortic ring ~15 mm).

### 2.8. Endothelium-Dependent Vasorelaxation of Thoracic Aortas

One of the ring segments was mounted in 5 mL organ bath filled with Tyrode's solution (37°C, gassed with 95% O_2_ and 5% CO_2_ mixture) under an optimal load of 1.2 g for 60 min. Changes in force were recorded by isometric force-displacement transducers connected to a PowerLab data acquisition system, where data sampling rate was set at 40 per minute. The rings were allowed to equilibrate for 60 min under their resting tension (1.2 g). During the equilibration period, the aortic rings were washed with drug-free Tyrode's solution every 20 min and the resting tension was readjusted, whenever necessary, before commencing the experiments. After the equilibration, the aortic rings were challenged with 60 mM KCl twice to sensitize the preparations. The contractile response (isometric tension, in g) was measured. To investigate the relaxant effects of acetylcholine on isolated aorta, the preparations were precontracted with phenylephrine (1 *μ*M) in the presence of indomethacin (1 *μ*M, a nonselective cyclooxygenase inhibitor) and neostigmine (1 *μ*M, an anticholinesterase). After a steady-state contraction was established, cumulative concentrations (10 nM–10 *μ*M) of acetylcholine were added to the organ bath. Concentration-response curves were plotted as percentage relaxation of phenylephrine-induced contraction against logarithmic concentration of acetylcholine.

### 2.9. *In Vitro* NO Production in the Isolated Thoracic Aortas

To evaluate the endothelial damage in blood vessels caused by high-cholesterol diet, *in vitro* production of nitrite (NO_2_^−^), which is one of two primary, stable, and nonvolatile breakdown products of NO, in the aortic ring was tested to estimate NO production. Briefly, the isolated aortas were washed twice with Tyrode's solution and then cut into 15 mm segments (weight = 0.03-0.04 g). The segments were incubated in a 24-well plate (containing 2 mL Tyrode's solution per well) with acetylcholine (1 *μ*M) and neostigmine (1 *μ*M). After incubation (37°C) for 2 h, each segment was blotted dry and weighed and the incubated culture solution of each well was collected in a separate microcentrifuge tube. The solution of each tube was subsequently dried by vacuum freeze-drying and the resulting pellets were redissolved with distilled water (300 *μ*L). Nitrite content was measured using the Griess reagent system (Promega, USA). The absorbance was determined using a spectrophotometer at 540 nm. The concentrations of nitrite were calculated following instructions of the kit.

### 2.10. Real-Time Polymerase Chain Reaction Analysis

Isolated thoracic aortas and livers were homogenized and total RNA was extracted using Trizol reagent (Invitrogen) for the determination of gene expression levels of aortic eNOS and hepatic PEPCK, LDLR, PPAR*α*, and HMGR in various groups. 2.0 *μ*g of total RNA was reverse transcribed into cDNA using RevertAid first-strand cDNA synthesis kit (Fermentas). Real-time PCR was performed with iQ SYBR Green Supermix (Bio-Rad) in 20 *μ*L of total reaction mixture. The primers for eNOS, PEPCK, LDLR, PPAR*α*, HMGR, and GAPDH were synthesized by Shanghai GeneCore Bio Technologies Co. Ltd. (China) (Table 1). Each real-time RT-PCR reaction was performed in triplicate and a standard curve was generated each time for assessing the expression level in cDNA. To correct for differences in quantity and quality between different RNA samples, the expression levels of various mRNA of interest were expressed as the ratios of mRNA expression of individual gene to that of the housekeeping gene, GAPDH, in the corresponding samples.

### 2.11. Western Blot Immunoreactivity Assay

Hepatic LDLR, PEPCK, PPAR*α*, and HMGR protein levels were quantified using immunoblotting procedures. Protein extracts (5 *μ*g for PEPCK and GAPDH, 20 *μ*g for LDLR and HMGR, 40 *μ*g for PPAR*α*) were applied to 12% SDS polyacrylamide gels and transferred to polyvinylidene difluoride membranes, which were blotted overnight at 4°C with nonfat dry milk solution. Blots were incubated with the appropriate antibody (Abcam Inc., USA) for 2 h at room temperature. The membrane was subsequently probed with a secondary goat anti-rabbit or goat anti-mouse IgG conjugated to horseradish peroxidase for 1 h at room temperature at a dilution of 1 : 5000 and 1 : 10000, respectively (Kangchen Bio-tech, Shanghai, China). Immunoreactive bands were visualized by Immun-Star WesternC Chemiluminescent Kit in a ChemiDoc XRS machine and analyzed using Quantity One 4.6.7 for Windows (Bio-Rad, U.S.A.) software. Each reported value was derived from the ratio between arbitrary units obtained by protein band and the respective GAPDH band (36 kDa, chosen as housekeeping protein).

### 2.12. Statistical Analysis

Data were expressed as means ± SEM and *n* denotes the number of replications for each data point. After validation of each parameter for homogeneity of variance, intergroup differences were analyzed by using one-way analysis of variance (ANOVA) and Dunnett's multiple comparison test as posttest after ANOVA was performed to compare the group means by the statistical software, GraphPad Prism 5.02 (San Diego, CA, USA) for Windows. A value of probability (*P*) < 0.05 was considered statistically significant.

## 3. Results

### 3.1. HPLC Fingerprint

**Figure 1:**
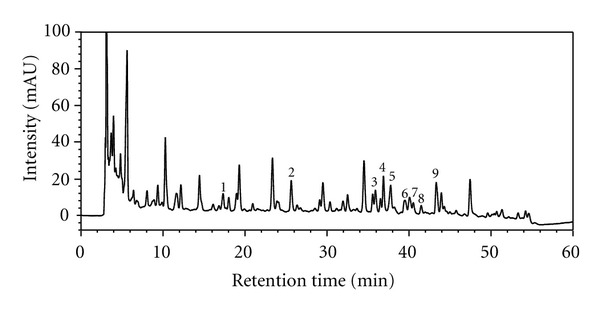
HPLC/UV (254 nm) chromatogram of the turtle jelly sample used in the current study. (1) caffeoylquinic acid, (2) 5-O-caffeoylshikimic acid, (3) astilbin, (4) 3,4-dicaffeoylquinic acid, (5) 4,5-dicaffeoylquinic acid, (6) neoisoastilbin, (7) isoastilbin, (8) engeletin, and (9) 3,5-dicafeoyylquinic acid.

A representative HPLC chromatogram was shown in Figure 1. Totally, nine peaks were identified from the sample by the mass spectrometer. Their identities and relative content (in terms of peak area under the 254 nm detection wavelength) in the freeze-dried GLG sample were caffeoylquinic acid (1.04%), 5-O-caffeoylshikimic acid (5.44%), astilbin (2.66%), 3,4-dicaffeoylquinic acid (2.02%), 4,5-dicaffeoylquinic acid (1.66%), neoisoastilbin (3.01%), isoastilbin (3.61%), engeletin (1.07%), and 3,5-dicafeoyylquinic acid (1.61%). 

### 3.2. Effects on Serum Lipids Profiles

**Figure 2:**
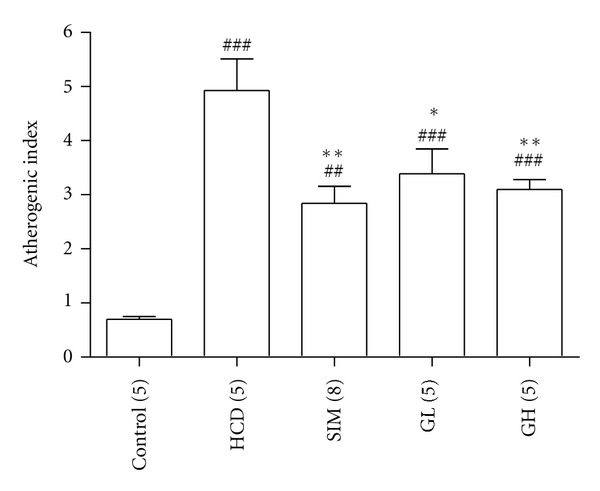
The atherogenic indexes of animals from the groups of Control, HCD, SIM (3 mg/kg bw per day), GL (3.3 mL/kg bw per day), and GH (10 mL/kg bw per day). Data are expressed as means ± SEM, *n* = 5–7. ^##^ *P* < 0.01 and ^###^ *P* < 0.001 represent significant differences when compared with the Control. *P < 0.05 and **P < 0.01 represent significant differences when compared with the HCD. The number in parentheses is *n* for individual group.

The lipid profiles: serum total cholesterol, triglycerides, HDL, and LDL from various rat groups are summarized in Table 2. A high-cholesterol diet significantly upregulated total cholesterol and LDL content in serum (both, *P* < 0.001). The serum total cholesterol was significantly reduced in the SIM (6.10 ± 0.81 mmol/L, *P* < 0.05), GH group (6.95 ± 0.65 mmol/L, *P* < 0.05) as compared with that in the HCD group (10.33 ± 1.05 mmol/L). Animals treated with simvastatin (3 mg/kg bw per day) (1.92 ± 0.26 mmol/L, *P* < 0.01) or turtle jelly (3.3 mL/kg bw per day: 2.45 ± 0.43 mmol/L, *P* < 0.05 or 10 mL/kg bw per day: 2.46 ± 0.28 mmol/L, *P* < 0.05) could significantly reduce serum LDL as compared with the HCD group (3.68 ± 0.37 mmol/L). However, both total cholesterol and LDL content in serum did not return to normal (control) level (total cholesterol: 2.30 ± 0.13 mmol/L; LDL: 0.52 ± 0.02 mmol/L) in all treatment groups. Animals with high-cholesterol diet showed no significant changes in the level of triglycerides (*P* > 0.05) or HDL (*P* > 0.05). Further analysis of the impact of serum lipid profiles on the progression of atherosclerosis, the atherogenic index [(total serum cholesterol − HDL)/HDL] (which measures coronary heart disease risk), was calculated. It was found that elevated atherogenic index was observed in the HCD group (Figure 2). Consumption of turtle jelly could significantly decrease the atherogenic indexes in a dose-dependent manner (GL: *P* < 0.05; GH: *P* < 0.01). Turtle jelly showed an effect comparable to simvastatin on lowering elevated atherogenic index, suggesting that both simvastatin and turtle jelly possess atheroscleroprotective potential in the current experimental setting. 

### 3.3. Determination of the Antioxidative Enzyme Activities and MDA Content in Livers

**Figure 3:**
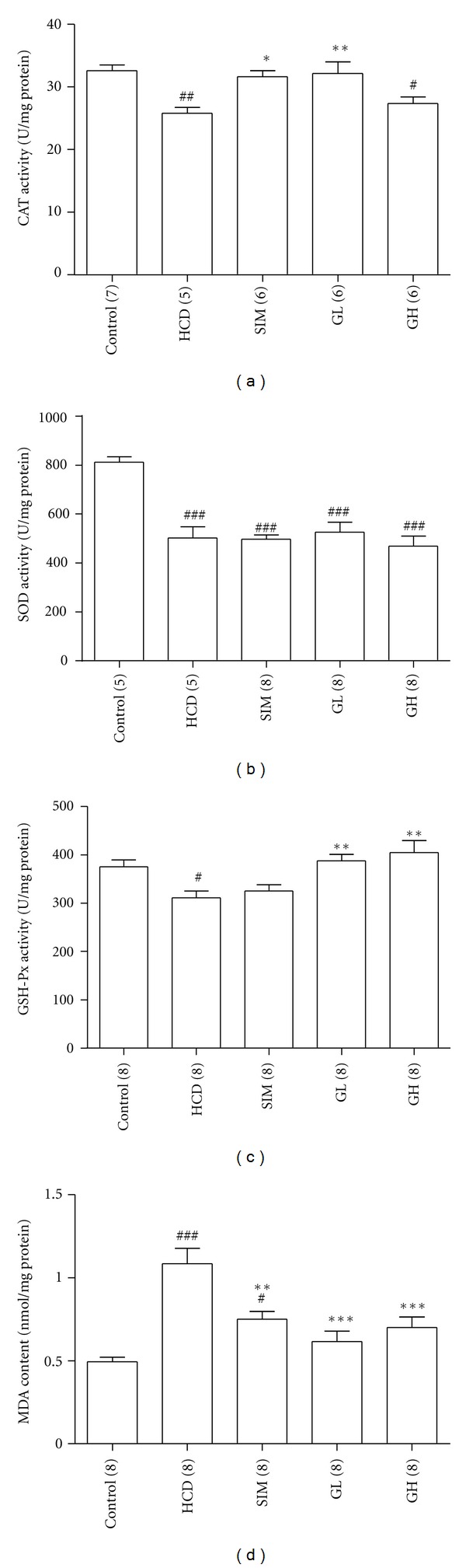
(a) CAT, (b) SOD, (c) GSH-PX activities, and (d) MDA contents of isolated livers from the Control, HCD, SIM (3 mg/kg bw per day), GL (3.3 mL/kg bw per day), and GH (10 mL/kg bw per day). Data are expressed as means ± SEM, *n* = 5–8. ^#^ *P* < 0.05, ^##^ *P* < 0.01, and ^###^ *P* < 0.001 represent significant differences when compared with the Control. *P < 0.05, **P < 0.01, and ***P < 0.001 represent significant differences when compared with the HCD. The number in parentheses is *n* for individual group.

Figure 3 depicts the activities of antioxidant enzymes: CAT, SOD, and GSH-Px in the livers of rats from the Control, HCD, SIM, GL, and GH groups. There was a marked decrease in CAT (*P* < 0.01), SOD (*P* < 0.01), and GSH-Px (*P* < 0.05) activities in the HCD group as compared with the control rats. For the activity of CAT, treating the animals with simvastatin (*P* < 0.05) or turtle jelly (3.3 mL/kg bw per day only, *P* < 0.01) managed to restore the high-cholesterol diet-induced reduction of CAT activity (Figure 3(a)). No significant changes (*P* > 0.05) in SOD activities were found amongst the HCD, SIM, GL, and GH groups as compared with the HDC group (Figure 3(b)). Comparing with the HCD rats, treating the animals with turtle jelly, but not simvastatin, could significantly raise the liver GSH-Px activity in a dose-dependent manner (*P* < 0.01, Figure 3(c)). A marked increase of MDA content in the HCD group was recorded as compared with the control rats (*P* < 0.001, Figure 3(d)). Treating the animals with simvastatin (*P* < 0.01) or turtle jelly (both 3.3 and 10 mL/kg bw per day, *P* < 0.001) significantly decreased the MDA content as compared with the HCD rats (Figure 3(d)).

### 3.4. Liver Histological Examination and Lipid Content Evaluation

**Figure 4:**
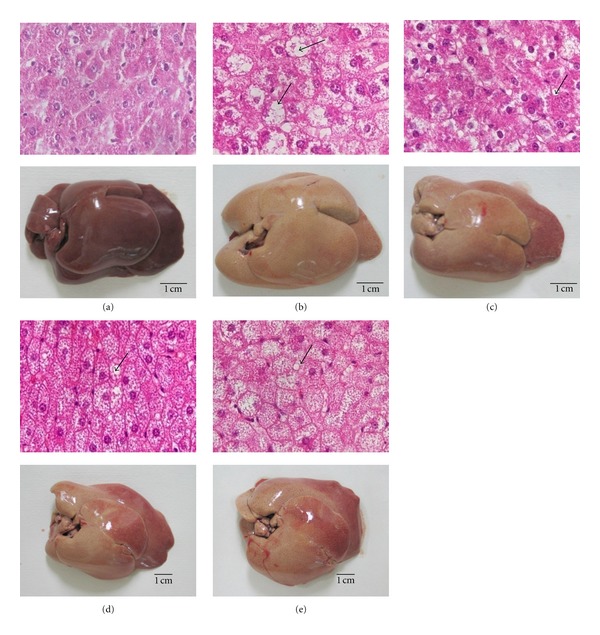
Histological examination of the livers obtained from the (a) Control, (b) HCD, (c) SIM (3 mg/kg bw per day), (d) GL (3.3 mL/kg bw per day), and (e) GH (10 mL/kg bw per day). Photographs of the cross-section (400× magnification) of livers (top) and the gross appearance of the entire livers (bottom) are illustrated. Arrows show the location of lipid depositions.

The gross appearances of the livers from rats fed with normal diet, high-cholesterol diet, high-cholesterol diet plus simvastatin or two different dosages of turtle jelly are depicted in Figure 4. The livers of the control (normal) rats had a relatively dark-red colour whereas those from the HCD group were enlarged with a yellowish colour. Treating the rats with simvastatin or turtle jelly could make the colour of liver relatively “less yellowish” as was observed in the HCD rats (Figures 4(c)–4(e)). Histological examination of the livers of the control rats revealed intact cellular structure (Figure 4(a)). In contrast, the livers of rats from the HCD group illustrated poor cellularity with extensive lipid depositions and enlarged hepatocytes (Figure 4(b)). In animals administrated with simvastatin or turtle jelly, a lesser degree of lipid deposition and hepatocytes enlargement was observed (Figures 4(c)–4(e)). 

**Figure 5:**
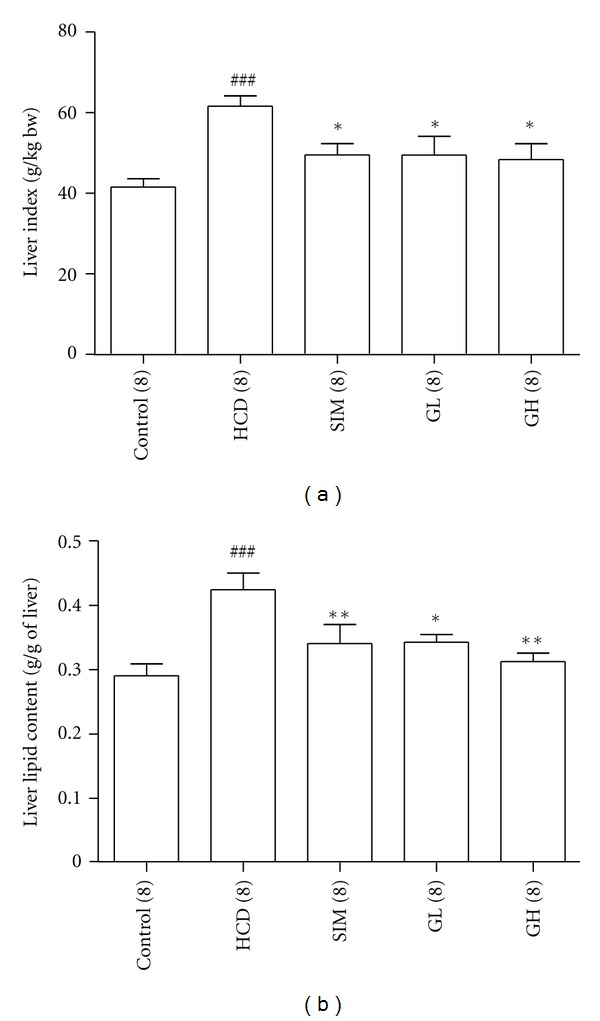
(a) Liver indexes (g of liver per kg bw) for various groups. (b) Total lipid contents (g of lipid per g of liver) isolated from various groups. Data are expressed as means ± SEM, *n* = 8. ^###^ *P* < 0.001 represents significant differences when compared with the Control. **P < 0.01 when compared with the HCD group.*P < 0.05 and **P < 0.01 represent significant differences when compared with the HCD. The number in parentheses is *n* for individual group.

In order to evaluate the lipid content in the livers from different treatment groups, (i) liver index (weight of liver (g) to animal's body weight (kg)) and (ii) total lipid in the liver was measured and compared (Figure 5). There were significant increases in both liver index (*P* < 0.001) and liver lipid content (*P* < 0.001) in the HCD group as compared with those in the Control group. Interestingly, supplementing the animals with 3.3 or 10 mL/kg bw per day of turtle jelly could dose-dependently reduce the increased liver index and lipid content of hypercholesterolemic rats (Figures 5(a) and 5(b)). This suggested the hepatoprotective effect of turtle jelly in the current experimental setting. Simvastatin exhibited hepatoprotective effect similar to turtle jelly.

### 3.5. Measurement of the Isometric Tension of the Isolated Thoracic Aortas

**Figure 6:**
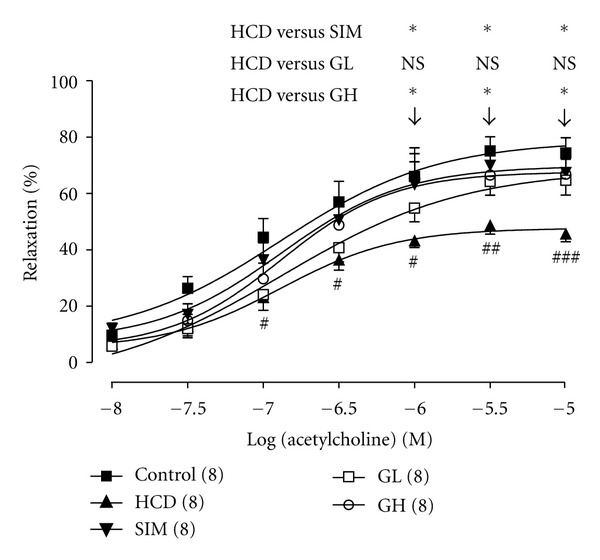
The concentration-response curves to acetylcholine are expressed as decrease in (percentage) steady-state tension obtained with 1 *μ*M phenylephrine precontracted thoracic aortic rings from the Control, HCD, SIM (3 mg/kg bw per day), GL (3.3 mL/kg bw per day), and GH (10 mL/kg bw per day). Data are expressed as means ± SEM, *n* = 8. ^#^ *P* < 0.05  ^##^ *P* < 0.01, and ^###^ *P* < 0.001 represent significant differences when compared with the Control. *P < 0.05 represents significant differences when compared with the HCD. The number in parentheses is *n* for individual group.

To evaluate the protective effect of turtle jelly administration on vascular endothelial activity, the thoracic aorta (with intact endothelium) was isolated for isometric tension analyses. When phenylephrine (1 *μ*M)-induced contraction reached a steady condition, acetylcholine was added cumulatively to the aortic preparation in the presence of neostigmine (1 *μ*M). Acetylcholine elicited a concentration (10 nM–10 *μ*M)-dependent aortic relaxation of various groups of rats with ~45–75% maximum relaxation at 10 *μ*M (*n* = 8) (Figure 6). A significantly smaller magnitude of relaxation caused by acetylcholine was observed in aortas from HCD rats as compared with those observed in other groups (*P* < 0.01) (Figure 6). Turtle jelly (3.3 or 10 mL/kg bw per day, *P* < 0.05) dose-dependently improved the acetylcholine-induced relaxation in rats with high-cholesterol diet. Ameliorations in the acetylcholine-induced relaxation were only observed in animals treated with simvastatin (3 mg/kg bw per day) or higher dose of turtle jelly (10 mL/kg bw per day) (Figure 6). 

### 3.6. *In Vitro* Nitrite Production and eNOS mRNA Expression Level in the Isolated Thoracic Aortas

**Figure 7:**
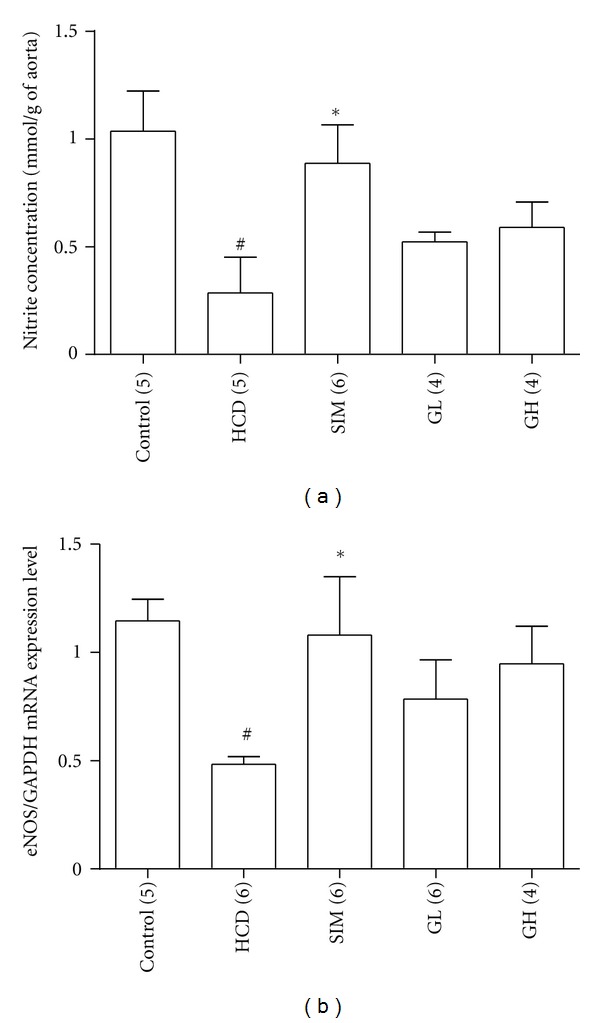
(a) *In vitro* nitrite productions from various groups' isolated aortas under challenge with acetylcholine (1 *μ*M) and (b) eNOS mRNA expressions in various groups' isolated aortas. The expression level of eNOS was normalized to that of the GAPDH. Data are expressed as means ± SEM, *n* = 4–6. ^#^ *P* < 0.05 represents significant differences when compared with the Control. *P < 0.05 represents significant differences when compared with the HCD. The number in parentheses is *n* for individual group.

The effect of turtle jelly treatment on high-cholesterol diet-induced damage on aortic endothelial cells was also evaluated. The isolated aortic rings from various groups were tested with regard to their nitrite production ability. Without acetylcholine (1 *μ*M), aortic rings released undetectable levels of nitrite after 2 h of incubation (data not shown). When acetylcholine (1 *μ*M) was added to the incubation medium, nitrite production in the Control group increased dramatically to 1.04 ± 0.19 mmol/g of aortic tissue after the 2 h incubation period. The effect of acetylcholine on nitrite production could be abolished by the NOS inhibitor L-NAME (20 *μ*M) (data not shown). It was found that high-cholesterol diet markedly attenuated nitrite production (0.29 ± 0.18 mmol/g of aortic tissue, *P* < 0.05). As shown in Figure 7(a), aortas from the simvastatin (3 mg/kg bw per day) treatment group significantly potentiated nitrite production and completely restored the HCD-induced vascular damage. There was a slight and insignificant increase in the turtle jelly treatment groups (both dosages) as compared with the HCD group (*P* > 0.05) (Figure 7(a)).

 Apart from estimating NO production (using nitrite production), changes in the mRNA expression of eNOS in aortas were also examined by quantitative real-time RT-PCR (Figure 7(b)). The mRNA expression level of eNOS in the aortas from the HCD group was markedly suppressed (*P* < 0.05); whereas treatment of simvastatin (3 mg/kg bw per day) could significantly increase eNOS expression level back to the level in the Control group (*P* < 0.05). Animals treated with turtle jelly (3.3 or 10 mL/kg bw per day) showed a slight and insignificant enhancement in the gene expression of eNOS (*P* > 0.05). The pattern of eNOS mRNA expressions observed amongst different groups was similar to the pattern of nitrite productions amongst the different groups (Figure 7). 

### 3.7. Effects of Turtle Jelly on Gene and Protein Expressions of LDLR, PEPCK, PPAR*α*, and HMGR in the Livers

**Figure 8:**
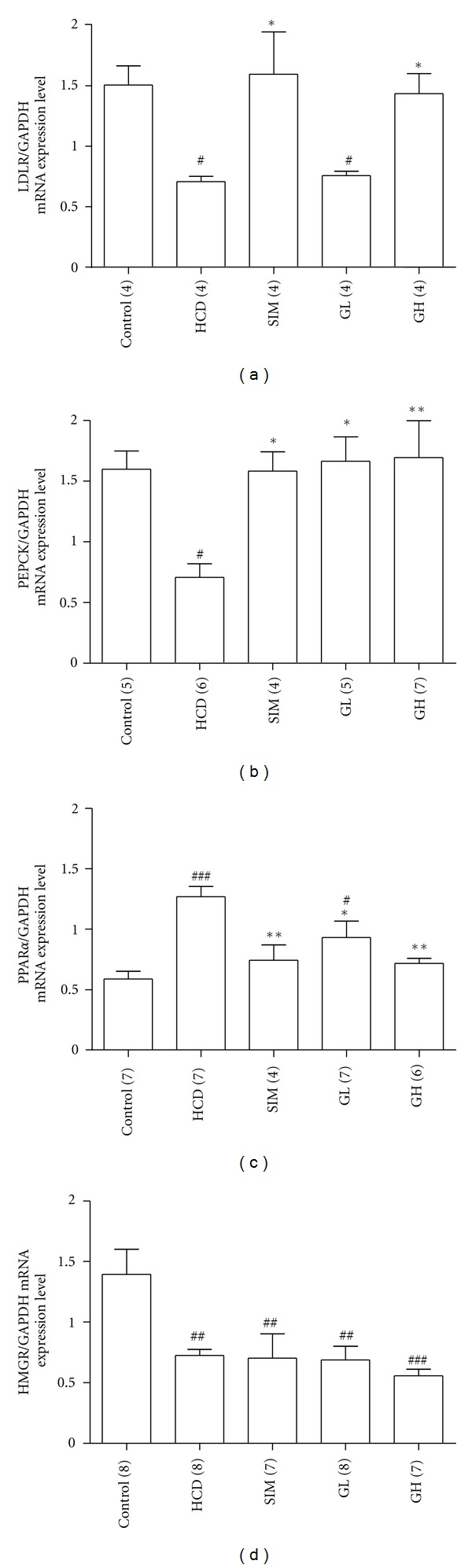
Real-time RT-PCR analysis of the mRNA expressions of (a) LDLR, (b) PEPCK, (c) PPAR*α*, and (d) HMGR in the livers from the Control, HCD, SIM (3 mg/kg bw per day), GL (3.3 mL/kg bw per day), and GH (10 mL/kg bw per day). The expression level of each gene was normalized to that of the GAPDH gene in each sample. Data are expressed as means ± SEM, *n* = 4–7. ^#^ *P* < 0.05, ^##^ *P* < 0.01, and ^##^ *P* < 0.01 represent significant differences when compared with the Control group. *P < 0.05 and **P < 0.01 represent significant differences when compared with the HCD. The number in parentheses is *n* for individual group.

**Figure 9:**
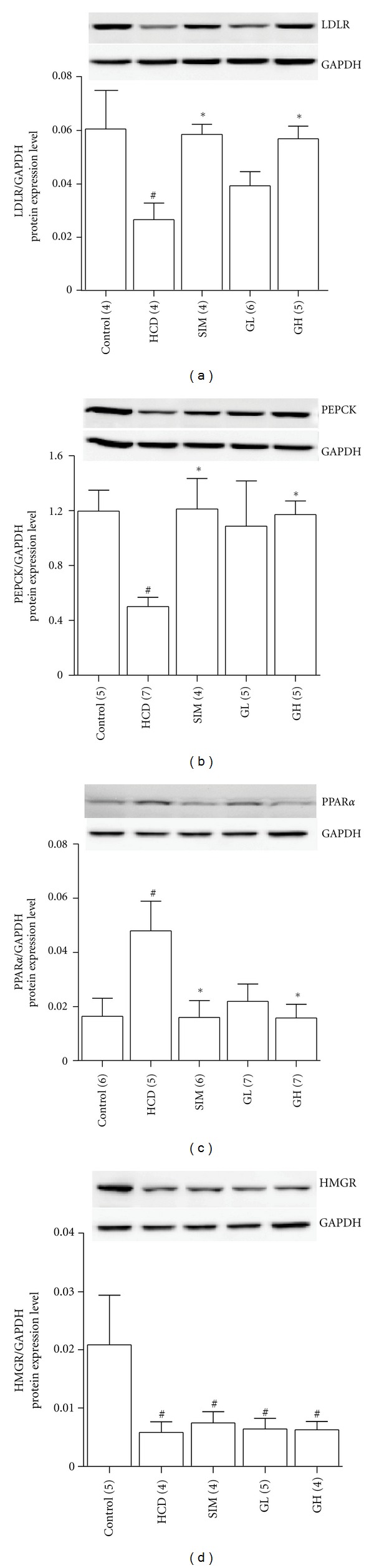
Representative Western blots and graphs represent quantitative comparisons of protein expressions of (a) LDLR, (b) PEPCK, (c) PPAR*α*, and (d) HMGR in the livers from the Control, HCD, SIM (3 mg/kg bw per day), GL (3.3 mL/kg bw per day), and GH (10 mL/kg bw per day). The expression level of each protein was normalized to that of the GAPDH protein in each sample. Data are expressed as means ± SEM, *n* = 4–7. ^#^ *P* < 0.05 represents significant differences when compared with the Control. *P < 0.05 represents significant differences when compared with the HCD. The number in parentheses is *n* for individual group.

Changes in the mRNA and protein expressions of LDLR, PEPCK, PPAR*α*, and HMG in the livers are shown in Figures 8 and 9. High-cholesterol diet could markedly suppress the gene and protein expression levels of LDLR (both expressions with *P* < 0.05, Figures 8(a) and 9(a)), PEPCK (both expressions with *P* < 0.05, Figures 8(b) and 9(b)) and HMGR (gene expression with *P* < 0.01, Figure 8(d); protein expression with *P* < 0.05, Figure 9(d)) but promote those of PPAR*α* (gene with *P* < 0.001, Figure 8(c); protein with *P* < 0.05, Figure 9(c)) in the livers as compared with their corresponding controls. Simvastatin (3 mg/kg bw per day) significantly restored (*P* < 0.05) the high-cholesterol diet-induced downregulations of LDLR and PEPCK mRNA and protein back to a level close to their normal conditions. The liver PPAR*α* mRNA (*P* < 0.01) and protein (*P* < 0.05) expressions were markedly suppressed in animals from the SIM group as compared with the HCD groups. Administration of turtle jelly led to a dose-dependent restoration of the mRNA and protein expressions of LDLR, PEPCK, and PPAR*α* in the livers (Figures 8 and 9). High dosage of turtle jelly (10 mL/kg bw per day) significantly reversed the high-cholesterol diet-induced changes in the mRNA and protein expressions of LDLR (both expressions with *P* < 0.05, Figures 8(a) and 9(a)), PEPCK (gene expression with *P* < 0.01, Figure 8(b); protein expression with *P* < 0.05, Figure 9(b)) and PPAR*α* (gene expression with *P* < 0.01, Figure 8(c); protein expression with *P* < 0.05, Figure 9(c)) in the livers as compared with their corresponding HCD groups. Both simvastatin and turtle jelly had no significant (*P* > 0.05) effects on the altering of the gene and protein expressions of HMGR as compared with the HCD animals (Figures 8(d) and 9(d)). 

## 4. Discussion

Diet plays an important role in the regulation of cholesterol homeostasis. In recent decades, functional food and natural dietary substances have been extensively studied to determine their role in preventing CVD [4, 7, 9]. In fact, hypercholesterolemic condition is regarded as an important factor in the development of CVD [10]. Therefore, natural products or functional foods with hypolipidemic and/or hypocholesterolemic effects are believed to be useful in reducing the risk of developing CVD [4]. In the present study, it has been demonstrated for the first time that high-cholesterol-induced complications such as increased lipid levels, fatty liver as well as endothelial dysfunction can be dose-dependently normalized by oral administration of turtle jelly at the dosage of 3.3 or 10 mL/kg bw per day for 30 days. The overall beneficial effects provided by high dosage of turtle jelly were better than those given by low dosage of turtle jelly. Although the effect of turtle jelly was not as good as simvastatin, a potent hypocholesterolemic and hypolipidemic drug, turtle jelly is still a good alternative to counteract the detrimental effects brought about by high-cholesterol diet.

 In the current study, the animals in the HCD group had abnormal serum lipid levels with significantly increased total cholesterol and LDL but no significant changes in triglyceride and HDL levels. These results are consistent with our pervious studies with similar experimental settings [7, 9]. The degree of elevation in serum total cholesterol and LDL levels is considerable enough to induce the atherogenic effect of the current model since both parameters play a major role in atherosclerosis development and the subsequent coronary heart disease [11]. We demonstrated that administration of turtle jelly significantly reduced serum total cholesterol (*P* < 0.05), LDL (*P* < 0.05), and LDL/HDL ratio (*P* < 0.05) in rats fed with high-cholesterol diet. The effects of high dosage of turtle jelly on the lipid profile were close to those given by simvastatin (3 mg/kg bw per day), a potent drug of statin series (such as lovastatin, fluvastatin, and pravastatin). Additionally, the elevated atherogenic index induced by high-cholesterol diet was suppressed significantly by turtle jelly. This suggested that turtle jelly consumption could provide atheroscleroprotective effect to the body.

 The liver is the primary organ to metabolize the cholesterol ingested. In the present study, extensive fat depositions were observed in the liver of rats fed with high-cholesterol diet alone. Histological examination showed that animals from the HCD group had enlarged livers (with higher liver index), accumulation of lipid depositions and overall lipid contents in the livers, loss of hepatocytes integrity, and hepatocytes enlargement. These changes are probably associated with or responsible for the aberrant changes of cholesterol/lipoprotein observed (i.e., fatty liver) in hypercholesterolemic rats. Turtle jelly is said to be capable of clearing toxicants from the blood, protecting the liver, and minimizing the effects of damp-heat [6]. The effects of turtle jelly on lowering serum cholesterol in blood and reducing lipid deposition in the liver could be interpreted as clearing toxicants from the blood and protecting the liver, respectively. Therefore, the current findings could be considered as strong scientific evidence for the anecdotal health benefit claims of consuming turtle jelly.

Hypercholesterolemic diet could affect the functions of liver substantially because of the high-cholesterol condition that causes an excessive production of reactive oxygen species (ROS), which in turn could initiate lipid peroxidation, damage liver functions, and affect the cardiovascular system [12–14]. Therefore, controlling the production of endogenous prooxidant levels in liver cells is important to prevent the development of CVD such as atherosclerosis [4, 15, 16]. Liver contains enzymes such as CAT, SOD, and GSH-Px which contribute to the antioxidant defense mechanisms [17]. Studies have shown that hypercholesterolemia diminishes the effectiveness of the antioxidant defense system and decreases the activities of CAT and SOD in rats [18, 19]. In the current experimental settings, decreases in CAT, SOD, and GSH-Px activities were observed in hypercholesterolemic rats as compared with those of the Control group. Administration of turtle jelly for 30 days to rats fed with high-cholesterol diet significantly elevated the activities of CAT and GSH-Px, but not the activity of SOD, in the liver. The mechanism for upregulation of CAT- and GSH-Px-specific enzyme activity induced by turtle jelly is current unknown and warrants further studies.

MDA, the product of lipid peroxidation, is an estimator of oxygen-free radical level. A decrease in lipid peroxidation leads to a reduction of atherosclerosis caused by hypercholesterolemia [20]. The content of MDA in rats fed with high-cholesterol diet was raised compared with the control animals, suggesting that hypercholesterolemia could enhance the process of lipid peroxidation. The oral administration of simvastatin or turtle jelly prevented a high-cholesterol diet-induced elevation of MDA and resulted in a significant decreased MDA content in the liver. These data suggested that turtle jelly could improve the efficiency of H_2_O_2_ reduction into H_2_O due to the increased GSH-Px and/or CAT activities as well as lower or slow down oxidative-stress-related lipid peroxidation.

Hypercholesterolemia and atherosclerosis are closely related to vascular dysfunction [21] because under hypercholesterolemic condition, endothelial production of superoxide, and possibly other ROS, increases and quenches NO [22]. NO is derived from endothelium which plays an important role in the homeostasis of vascular tone and structure under normal circumstances [23]. In the current study, a blunted acetylcholine-induced vascular relaxation which represents an endothelial/NO dysfunction was found in the aortas isolated from the HCD group. This is consistent with previous reports that described in animals [7, 24] and humans [25]. Simvastatin improved acetylcholine-induced relaxation on isolated aortic rings, an improvement that can be explained by the upregulation of eNOS mRNA expression and/or increase in acetylcholine-induced nitrite production (represented an increase in NO production) that counteract NO inactivation under hypercholesterolemic condition. It was found that the consumption of turtle jelly for 30 days by hypercholesterolemic rats could dose-dependently prevent endothelial/NO dysfunction with slight (but statistically insignificant) enhancements in both eNOS gene expression and NO production. The observed statistically insignificant phenomenon might be due to the limited sample size and/or short treatment period. Further studies with increased number of animals per group and/or longer treatment period could, undoubtedly, help give a conclusive explanation on the mechanistic pathway of turtle jelly in the prevention of endothelial dysfunction induced by hypercholesterolemic diet. 

The effects of turtle jelly on the expressions of LDLR, PEPCK, PPAR*α*, and HMGR mRNA and proteins in the liver were also under investigation in hypercholesterolemic animals treated with turtle jelly. The liver is the most important site for cholesterol metabolism. HMGR is an enzyme responsible for cholesterol synthesis. It is believed that HMGR is regulated through negative feedback of cholesterol [9]. Low cholesterol level would trigger HMGR transcription so as to enhance cholesterol synthesis. In this study, suppressed mRNA and protein expressions of HMGR were observed in the HCD, SIM, GL, and GH groups. There were no significant differences in HMGR (mRNA and protein) expressions amongst the four groups of high-cholesterol diet-fed animals. Hence the hypocholesterolemic effect exerted by turtle jelly may not be related to the inhibition of cholesterol synthesis.

LDL (the “bad” cholesterol) is closely associated with CVD. The LDLR pathway mediates at least 60–75% of LDL turnover in rats [26, 27] and 56–80% in humans [26, 28]. Since the liver contains about 70% of total LDLR present in the body [29], changes in serum LDL levels are generally due to changes in hepatic LDLR activity. In this study, hepatic LDLR expression was investigated. We found that hypercholesterolemic diet caused a significant reduction in hepatic LDLR mRNA and protein expression levels. Simvastatin (3 mg/kg bw per day) and turtle jelly (10 mL/kg bw per day) could significantly upregulate both LDLR mRNA and protein expressions, which in turn reduced the serum LDL level. In fact, similar observations about simvastatin treatment on LDLR had been reported in other studies [30–32], but this report is the first about the upregulation of both LDLR mRNA and protein expressions by turtle jelly. It is interesting to note that hepatic LDLR mRNA and protein expressions were only enhanced significantly in animals in the SIM and GH groups, (but not GL); whiles all three treatment groups (SIM, GL, and GH) could markedly lower serum LDL. This mismatch might indicate that turtle jelly could provide LDL-lowering effect through more than one pathway. High dosage of turtle jelly (10 mL/kg bw per day) predominately adopted upregulating LDLR to facilitate its LDL-lowering effect, but low dosage of turtle jelly (3.3 mL/kg bw per day) did not. Confirmatory studies on other animal models are warranted. In order to explore the mechanism(s) of turtle jelly on lowering serum cholesterol and LDL, further studies that include the monitoring on fecal lipid or fecal bile acid excretion are warranted. 

PEPCK is a rate-limiting enzyme in renal and hepatic gluconeogenesis. Hepatic PEPCK plays a crucial role in modulating lipid homeostasis and is regulated by a variety of dietary and hormonal signals [33]. Liver-specific PEPCK knockout mice exhibit impaired lipid metabolism and develop hepatic steatosis apparently because glyceroneogenesis is an important function of PEPCK in hepatocytes [34, 35]. In this study, the PEPCK gene and protein expression levels were significantly decreased in the HCD group, which showed obvious fatty and enlarged liver, accumulation of lipid deposition around hepatocyes and increase in overall hepatic lipid content as compared with those of the Control group. Similar results had been reported by others [36]. Treating hypercholesterolemic rats with turtle jelly markedly elevated PEPCK gene and protein expressions as compared with the Control group. Turtle jelly could promote the generation of glucose from noncarbohydrate carbon substrates such as glycerol from lipid by regulating the PEPCK pathway. Therefore, it could signal the body to use more lipid and/or cholesterol as the energy source so as to give its hypocholesterolemic effect.

To further understand the role of turtle jelly in lipid homeostasis, the mRNA and protein expressions of PPAR*α* were also investigated. PPAR*α*, a ligand-activated transcription factor, is primarily expressed in the liver and is known to promote the *β*-oxidation of fatty acids and play a pivotal role in the regulation of lipid homeostasis [37, 38]. It has been shown that mice lacking PPAR*α* exhibit hepatic steatosis, hypoglycemia, and myocardial lipid accumulation [39]. PPAR*α* mRNA expression was positively correlated with dietary fat intake in rat as an increased supply of fatty acids, endogenous ligand for PPAR*α* receptor, might contribute to an increased PPAR*α* signaling and upregulated PPAR*α* mRNA expression [40]. This can explain why agonists of PPAR*α* have atheroscleroprotective effects. In the current experimental condition, animals in the HCD group showed an upregulation of PPAR*α* mRNA and protein expressions. This might be due to the extra 5.5% oil in the diet which provided more fatty acids to stimulate PPAR*α* receptors in the liver. The amelioration of the upregulated PPAR*α* gene and protein expressions induced by hypercholesterolemic diet was achieved in animals treated with simvastatin or turtle jelly (10 mL/kg bw per day). Simvastatin and turtle jelly may have direct effect on the PPAR*α* mRNA and protein expressions in the liver. However, the possibility that the observed downregulation of PPAR*α* mRNA and protein expressions in the treatment groups is solely due to the hypocholesterolemic effect through other physiological pathways cannot be ruled out. A study of the role of turtle jelly in the PPAR*α* signaling pathway is being carried out in our laboratory to explain the hypocholesterolemic and hepatoprotective effects of turtle jelly.

## 5. Conclusions

The present results demonstrated for the first time that consumption of turtle jelly for 30 days has significant hypocholesterolemic effect in terms of reducing the plasma total cholesterol and LDL and alleviating the plasma HDL in rats fed with high-cholesterol diet. For the prominent improvements on atherogenic index after treating hypercholesterolemic rats with turtle jelly, turtle jelly was suggested to possess antiatherogenic capacity. In addition, turtle jelly administration could demonstrate good hepatoprotective function in terms of reducing fat depositions and overall lipid contents in the liver, increasing the activities of hepatic antioxidative enzymes such as GSH-Px and CAT activities and lowering oxidative-stress-related lipid peroxidation. The blunted NO/endothelium-mediated aortic relaxation in rats fed with high-cholesterol diet was restored to a certain extent after turtle jelly consumption. It is postulated that hypocholesterolemic effect is the primary beneficial effect given by turtle jelly and it leads to other secondary beneficial effects such as vasoprotective and hepatoprotective functions. The present data suggest that turtle jelly could block the downregulation of LDLR and PEPCK mRNA and protein expressions as well as suppress the upregulation of PPAR*α* mRNA and protein expressions in the livers in rats fed with high-cholesterol diet. Although the precise molecular mechanism exerted by turtle jelly on regulating lipid and cholesterol metabolisms is currently unknown, the present study provides clear scientific evidence to prove the anecdotal health-benefit claims that the consumption of turtle jelly as a functional food could clear toxicants (such as cholesterol and LDL) from the blood and protect the liver. Further studies on other animal models as well as other cholesterol metabolism mechanisms would provide in-depth understanding of the hypocholesterolemic effects of turtle jelly.

## Figures and Tables

**Table 1:** The primer sets used for real-time PCR.

Gene name	Forward primer (5′ to 3′)	Reverse primer (5′ to 3′)	Product size (bp)	NCBI reference sequence
eNOS	GGATTCTGGCAAGACCGATTAC	GGTGAGGACTTGTCCAAACACT	159	NM_021838.2
PEPCK	GCGGATACGGTGGGAACTCA	TGTCTTCACTGAGGTGCCCG	332	NM_198780.3
LDLR	TGGCTATGAGTGCCTATGTC	GGTGAAGAGCAGAAACCCTA	211	NM_175762
PPAR*α*	GCCGTTTCCACAAGTGCC	GCTAGTCTTTCCTGCGAGTATG	236	NM_013196.1
HMGR	CAACATCGTCACTGCCATC	GATGCTCAAGCTGCCTTCTT	411	X55286
GAPDH	TGCACCACCAACTGCTTAG	AGTGGATGCAGGGATGATGT	180	NM_017008

**Table 2:** Serum lipid levels of rats in various groups.

Animals groups	Control (*n* = 5)	HCD (*n* = 5)	SIM (*n* = 7)	GL (*n* = 5)	GH (*n* = 5)
Total cholesterol (mmol/L)	2.30 ± 0.13	10.33 ± 1.05^###^	6.10 ± 0.81^#,∗^	7.40 ± 1.12^##^	6.95 ± 0.65^##,∗^
Triglyceride (mmol/L)	3.26 ± 0.52	3.29 ± 0.29	2.26 ± 0.50	2.40 ± 0.46	2.98 ± 0.36
LDL (mmol/L)	0.52 ± 0.02	3.68 ± 0.37^###^	1.92 ± 0.26^##,∗∗^	2.45 ± 0.43^###,∗^	2.46 ± 0.28^###,∗^
HDL (mmol/L)	1.37 ± 0.11	1.74 ± 0.04	1.58 ± 0.12	1.66 ± 0.13	1.69 ± 0.13
LDL/HDL	0.39 ± 0.04	2.11 ± 0.20^###^	1.22 ± 0.13^###,∗∗∗^	1.44 ± 0.18^###,∗^	1.45 ± 0.10^###,∗^

Data are expressed as means ± SEM, (*n* = 5–7).

^#^ *P* < 0.05, ^##^ *P* < 0.01, and ^###^ *P* < 0.001 represent significant differences when compared with the control group.

*P < 0.05, **P < 0.01, and ***P < 0.001 represent significant differences when compared with the HCD group.

## Abbreviations

CVD: Cardiovascular diseases
HDL: High-density lipoprotein
LDL: Low-density lipoprotein
HCD: High-cholesterol diet fed group
SIM: High-cholesterol diet fed plus simvastatin (3 mg/kg bw per day, p.o.) treated group
GL: High-cholesterol diet fed plus low dose of turtle jelly (3.3 mL/kg bw per day, p.o.) treated group
GH: High-cholesterol diet fed plus high dose of turtle jelly (10 mL/kg bw per day, p.o.) treated group
HPLC: High-performance liquid chromatography
SOD: Superoxide dismutase
GSH-Px: Glutathione peroxidase
CAT: Catalase
MDA: Maleic dialdehyde
NO: Nitric oxide
eNOS: Endothelial nitric oxide synthase
PEPCK: Phosphoenol pyruvate carboxykinase
LDLR: Low-density lipoprotein receptor
PPAR*α*: Peroxisome proliferator-activator receptor *α*
HMGR: 3-Hydroxy-3-methyl-glutaryl-CoA reductase
GAPDH: Glyceraldehydes 3-phosphate dehydrogenase
ROS: Reactive oxygen species.
